# Exercise and the hepatic sirtuin network: rethinking the research focus on SIRT1, SIRT3, and SIRT6

**DOI:** 10.3389/fphys.2026.1755772

**Published:** 2026-01-14

**Authors:** Jun Woo Kwon

**Affiliations:** Sports Technology Laboratory, Department of Physical Education, Seoul National University, Seoul, Republic of Korea

**Keywords:** exercise, hepatic metabolism, mitochondrial function, NAFLD, sirtuins

## Abstract

Exercise remains one of the most effective non-pharmacological strategies for improving non-alcoholic and metabolic-associated fatty liver disease (NAFLD/MASLD). Beyond its systemic effects, regular physical activity rewires hepatic energy metabolism and enhances mitochondrial efficiency. Within this adaptive process, members of the Sirtuin family-particularly SIRT1, SIRT3 and SIRT6-have received considerable attention. These proteins operate at distinct regulatory layers: SIRT1 modulates transcriptional programs, SIRT3 shapes mitochondrial metabolic fluxes, and SIRT6 influences chromatin architecture and epigenetic repression. Together, they are widely regarded as the principal molecular mediators linking exercise to improved hepatic metabolic function. However, the Sirtuin family consists of seven members, and accumulating evidence indicates that the remaining isoforms-SIRT2, SIRT4, SIRT5 and SIRT7-also participate in the hepatic response to exercise. Their potential roles include buffering metabolic stress, supporting protein quality control, and modulating inflammatory signaling, suggesting a broader regulatory network than currently emphasized. This Perspective revisits the exercise–Sirtuin axis from a mechanistic physiology standpoint. It examines why research focus has historically converged on SIRT1, SIRT3 and SIRT6, and considers emerging data implicating the full Sirtuin repertoire in exercise-induced metabolic remodeling. The argument put forward is that future work may benefit from a more integrated framework that views exercise not as a trigger for a few dominant pathways, but as a stimulus capable of reorganizing an interdependent Sirtuin network governing hepatic metabolic resilience. Collectively, these considerations prompt a shift from a single-axis understanding toward a distributed regulatory model of hepatic adaptation to exercise.

## 1 Exercise reshapes hepatic NAD^+^ metabolism

Exercise serves as more than a means of expending calories; it represents a systemic physiological stimulus (Vargas-Ortiz et al., 2019). Across modalities-including high-intensity aerobic exercise, interval training, and resistance exercise-the hepatic NAD^+^/NADH ratio increases, forming the biochemical entry point for Sirtuin activation (Imai and Yoshino, 2013). During exercise, cellular energy depletion signals activate AMPK, which subsequently enhances NAD^+^ biosynthesis through the NAMPT pathway. This establishes the AMPK–SIRT1–PGC-1α signaling axis, a pathway that propagates its effects across nuclear, mitochondrial, and epigenetic regulatory layers (Ruderman et al., 2010; Su et al., 2024). Although most mechanistic insights into the hepatic NAD^+^–Sirtuin axis derive from rodent models, human exercise studies also support a link between training, NAD^+^ salvage, and Sirtuin activation. Endurance and resistance training increase NAMPT expression and NAD^+^ salvage capacity in human skeletal muscle, alongside higher SIRT1 and SIRT3 activity, suggesting that similar systemic signals may impinge on the liver during chronic exercise (Brandauer et al., 2013; Costford et al., 2010; Wasserfurth et al., 2021).

Within this framework, SIRT1, SIRT3, and SIRT6 operate as a coordinated three-tier module of the exercise response (Kanwal et al., 2019; Vargas-Ortiz et al., 2019). Through their sequential activation, exercise promotes a cascade that ultimately contributes to the attenuation of fatty liver pathology (Vargas-Ortiz et al., 2019).

**FIGURE 1:**
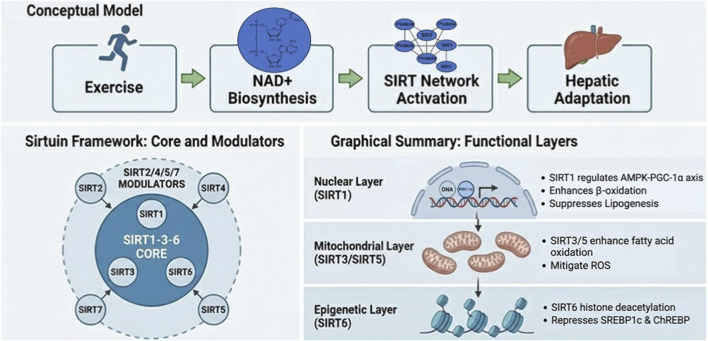
Integrated conceptual framework of exercise-induced hepatic adaptation mediated by the Sirtuin network **(A)** Exercise acts as a systemic physiological stimulus that enhances hepatic NAD^+^ biosynthesis, leading to activation of the Sirtuin network and subsequent hepatic adaptation. The NAD^+^ molecular structure shown in this panel is adapted from Wikimedia Commons and is licensed under the Creative Commons Attribution–ShareAlike 4.0 International License (CC BY-SA 4.0). **(B)** Within this network, SIRT1, SIRT3, and SIRT6 form a central functional core driving primary metabolic remodeling, while SIRT2, SIRT4, SIRT5, and SIRT7 function as modulators that fine-tune, stabilize, and protect the adaptive response. **(C)** Layer-specific actions of the Sirtuin network are illustrated across nuclear (SIRT1-mediated transcriptional regulation), mitochondrial (SIRT3/5-mediated enhancement of fatty acid oxidation and redox control), and epigenetic (SIRT6-mediated histone deacetylation and repression of lipogenic gene programs) levels, collectively contributing to exercise-induced hepatic remodeling.

This tiered organization is reflected not only in their activation patterns but also in their distinct functional roles across nuclear, mitochondrial, and epigenetic layers. At the nuclear level, SIRT1 modulates the AMPK–PGC-1α signaling axis to regulate transcription, suppress lipogenesis, and enhance β-oxidation (Anggreini et al., 2022; Cantó and Auwerx, 2009; Wu et al., 2022). At the mitochondrial level, SIRT3 enhances fatty acid oxidation and mitigates reactive oxygen species production through the deacetylation of key metabolic enzymes (Ansari et al., 2017; Bell and Guarente, 2011; Cheng et al., 2016; Trinh et al., 2024; Wu et al., 2022). At the epigenetic level, SIRT6 contributes to hepatic metabolic remodeling by repressing lipogenic transcription factors such as SREBP1c and ChREBP through histone deacetylation (Wu et al., 2022; Zhu et al., 2021). To provide a conceptual overview of how exercise-driven changes in NAD^+^ metabolism reorganize the hepatic Sirtuin network across nuclear, mitochondrial, and epigenetic layers, this framework is summarized in Figure 1.

## 2 Why does exercise research focus almost exclusively on SIRT1, SIRT3, and SIRT6?

The dominance of these three Sirtuins in exercise physiology literature does not stem solely from their presumed biological centrality. Rather, they are studied because their experimental behavior is clear, reproducible, and technically tractable (Vargas-Ortiz et al., 2019).

SIRT1 responds rapidly to both endurance exercise and high-intensity interval training, owing to its tight coupling with the AMPK–PGC-1α axis (Cantó and Auwerx, 2009; Imai and Yoshino, 2013; Ruderman et al., 2010). SIRT3 reacts almost immediately after exercise, as it is directly involved in mitochondrial biogenesis and reactive oxygen species regulation (Ansari et al., 2017; Cheng et al., 2016). Meanwhile, SIRT6 becomes more prominent during prolonged training, where its epigenetic effects help explain forms of metabolic “memory” (Kuang et al., 2018; Mostoslavsky et al., 2006).

Because these molecules yield consistent and interpretable results, attention-and consequently funding and publications-has accumulated around them. In other words, research has gravitated toward the Sirtuins that generate visible and stable outcomes (Pacifici et al., 2019).

Technical limitations have further reinforced this bias. Among the seven Sirtuin isoforms, viable and well-characterized genetically modified mouse lines exist primarily for SIRT1, SIRT3, and SIRT6. These models remain compatible with whole-body and liver-specific deletion strategies and display clear hepatic metabolic phenotypes under exercise and dietary intervention (Cheng et al., 2016; Kuang et al., 2018; Mostoslavsky et al., 2006). In contrast, SIRT2 and SIRT5 knockout mice show subtle or inconsistent liver phenotypes, while SIRT4 and SIRT7 deletions are associated with reduced viability, growth impairments, or reproductive defects-conditions that hinder long-term exercise-based protocols (Wu et al., 2022; Yoshizawa et al., 2014).

Thus, the current research landscape has been shaped less by the inherent functional importance of only three Sirtuins and more by the practical reality that only a subset is experimentally workable. The field, by necessity, has evolved around what can be studied rather than all that may be biologically relevant (Vargas-Ortiz et al., 2019; Wu et al., 2022).

This research imbalance aligns with differences in experimental tractability across Sirtuin isoforms. As shown in Tables 1, 2, the availability and robustness of genetic models have favored SIRT1, SIRT3, and SIRT6, whereas significant limitations remain for SIRT2, SIRT4, SIRT5, and SIRT7.

**TABLE 1:** Experimental basis for the research bias toward SIRT1, SIRT3, and SIRT6.

Sirtuin	Mouse model availability	Viability	Major phenotype	Suitability for NAFLD/Exercise studies
SIRT1	Global knockout, liver-specific knockout, and overexpression models available (Wang et al., 2011; Xu et al., 2010)	Viable (with mild growth impairment) Xu et al. (2010)	Insulin resistance, hepatic steatosis, inflammation (Wang et al., 2011; Xu et al., 2010; Ding et al., 2017; Zeng and Chen, 2022)	Highly suitable; compatible with both dietary and exercise intervention models (Pacifici et al., 2019; Ding et al., 2017)
SIRT3	Global and liver-specific knockout models available (Barroso et al., 2020; Kendrick et al., 2011)	Viable (Kendrick et al., 2011)	Impaired mitochondrial function, increased ROS, inducible fatty liver (Barroso et al., 2020; Kendrick et al., 2011)	Widely used mitochondrial metabolic model (Gibril et al., 2024; Pacifici et al., 2019)
SIRT6	Global knockout results in early mortality; liver-specific knockout (LKO) viable (Kuang et al., 2018; Mostoslavsky et al., 2006)	Viability preserved in tissue-specific model (Kuang et al., 2018; Zhong et al., 2020)	Premature aging and metabolic dysfunction; hepatic steatosis and insulin resistance (Kuang et al., 2018; Mostoslavsky et al., 2006; Xiao et al., 2012)	Actively used in studies, particularly regarding epigenetic regulation (Mei et al., 2016; Xiao et al., 2012; Zhong et al., 2020)

**TABLE 2:** Limitations of available experimental models for SIRT2, SIRT4, SIRT5, and SIRT7.

Sirtuin	Model characteristics	Limitations	Implications for NAFLD/Exercise research
SIRT2	Global knockout model available (Li et al., 2023; Lantier et al., 2018; Mei et al., 2016)	Viable but presents neurological abnormalities and extrahepatic defects, and shows context-dependent metabolic phenotypes across dietary and sex conditions (Lantier et al., 2018; Li et al., 2023; Mei et al., 2016; Schmidt et al., 2024)	Recent NAFLD models indicate that SIRT2 can influence hepatic steatosis and insulin resistance, but these effects remain heterogeneous and are often accompanied by pronounced systemic and neurological phenotypes, making SIRT2 less frequently used as a primary model in exercise-focused NAFLD research (Lantier et al., 2018; Li et al., 2023; Mei et al., 2016; Schmidt et al., 2024)
SIRT4	Global knockout results in metabolic dysregulation and reduced fertility (Mei et al., 2016; Nasrin et al., 2010)	Energy balance becomes unstable, hindering long-term intervention studies (Laurent et al., 2013; Mei et al., 2016)	Poor suitability for chronic fatty liver models (Laurent et al., 2013; Mei et al., 2016; Nasrin et al., 2010)
SIRT5	Global knockout viable but displays mild or subtle phenotypes (Mei et al., 2016)	Presence of compensatory pathways obscures clear metabolic effects (Goetzman et al., 2020; Mei et al., 2016)	Results in hepatic lipid studies remain inconsistent (Goetzman et al., 2020; Mei et al., 2016)
SIRT7	Global knockout associated with early mortality, growth delay, and muscle wasting (Mei et al., 2016; Shin et al., 2013; Yoshizawa et al., 2014)	Reduced survival and growth defects can limit the feasibility of prolonged dietary or exercise intervention protocols, and although tissue-specific and conditional models are emerging, their availability and use remain comparatively restricted (Mei et al., 2016; Shin et al., 2013; Tang, 2015; Yoshizawa et al., 2014; Yoshizawa et al., 2022)	Available models therefore provide important mechanistic insight into hepatic and systemic SIRT7 function, but their survival and growth constraints still restrict long-term NAFLD and exercise-intervention studies (Shin et al., 2013; Tang, 2015; Yoshizawa et al., 2014; Yoshizawa et al., 2022)

## 3 Exercise also influences SIRT2, SIRT4, SIRT5, and SIRT7

Recent findings increasingly suggest that exercise does not selectively activate only SIRT1, SIRT3, and SIRT6, but also induces measurable-though often subtler-changes in the remaining Sirtuin isoforms (Wu et al., 2022).

SIRT2 expression tends to rise alongside reductions in inflammatory cytokines such as IL-6 and TNF-α following exercise. This shift is linked to dampened hepatic macrophage activation and improved insulin sensitivity, positioning SIRT2 as a mediator of the exercise-associated anti-inflammatory response. Notably, direct exercise intervention studies using SIRT2-deficient models remain scarce, and current interpretations are largely inferred from metabolic and inflammatory phenotypes rather than confirmed exercise-specific experiments (Lantier et al., 2018). Recent diet-induced NAFLD models further suggest that SIRT2 deficiency can aggravate hepatic steatosis and insulin resistance, although these effects appear to depend on dietary context, sex, and gut microbiota composition (Li et al., 2023; Schmidt et al., 2024).

SIRT4 appears to function differently. By suppressing mitochondrial enzyme activity, it prevents excessive oxidative metabolism during exercise, thereby acting as a safeguard against metabolic overuse and contributing to cellular protection (Laurent et al., 2013; Nasrin et al., 2010).

Although direct evidence linking SIRT5 activity to exercise-induced mitochondrial adaptations in the liver remains limited, SIRT5-mediated desuccinylation has been shown to regulate mitochondrial enzyme efficiency and oxidative stress responses in metabolic tissues. These mechanisms may complement the well-established role of SIRT3 during exercise-induced mitochondrial remodeling, suggesting a potential cooperative relationship under conditions of increased energetic demand (Goetzman et al., 2020; Wu et al., 2022).

SIRT7 responds to exercise-induced translational demand. It contributes to relief of ER stress and supports ribosomal protein expression stability during prolonged adaptation, implying a role in maintaining cellular resilience when exercise becomes a chronic physiological load (Shin et al., 2013; Yoshizawa et al., 2014).

Viewed collectively, if SIRT1, SIRT3, and SIRT6 form the core pathway that generates the metabolic benefits of exercise, then SIRT2, SIRT4, SIRT5, and SIRT7 can be viewed as a complementary regulatory layer-one that fine-tunes, stabilizes, and protects the metabolic remodeling triggered by physical activity (Zeng and Chen, 2022).

## 4 Beyond the core SIRT1–SIRT3–SIRT6 triad: a post-triadic view of exercise physiology

The current framework in exercise physiology may need to move beyond the question of which Sirtuin becomes activated. A more relevant direction is to examine how the entire Sirtuin network is reorganized in response to exercise intensity, duration, and nutritional context. In this shift, the network-level behavior-not a single molecular response-becomes the central inquiry (Vargas-Ortiz et al., 2019).

Potential directions for future investigation include: 1) Applying single-cell transcriptomic approaches to identify cell-type–specific shifts in hepatic Sirtuin expression following exercise; 2) Using NAD^+^ flux tracing to define how interactions between SIRT1 and SIRT3 differ across exercise intensities; 3) Exploring diet–exercise combination models to clarify the stress-buffering roles of SIRT4 and SIRT5 (Wasserfurth et al., 2021); 4) Studies following these trajectories would allow exercise physiology to be interpreted not as a series of isolated protein responses but as a dynamic reconfiguration of metabolic circuitry (Han et al., 2019; Schmidt et al., 2024; Yoshizawa et al., 2022).

In addition, emerging questions remain regarding the dynamic regulation of epigenetic modifications beyond SIRT6, including whether other Sirtuin isoforms contribute to the establishment of metabolic memory following repeated exercise exposure. An equally intriguing but largely unexplored area concerns potential transgenerational effects, whereby exercise-induced reorganization of the hepatic Sirtuin network in parents may influence metabolic programming in offspring. This network-based view is consistent with broader systems-level analyses proposing that Sirtuins function as an interconnected metabolic control layer, coordinating energy homeostasis across multiple tissues rather than acting as isolated regulators (Chalkiadaki and Guarente, 2012; Houtkooper et al., 2012; Maissan et al., 2021).

## 5 Conclusion

Exercise reconfigures hepatic energy metabolism and can reverse fatty liver pathology, with SIRT1, SIRT3, and SIRT6 at the center of this response. Yet the physiological impact of exercise extends far beyond energy expenditure. Throughout this adaptive process, SIRT2, SIRT4, SIRT5, and SIRT7 modulate stress signaling, maintain protein quality control, and support cellular recovery-functions that help sustain long-term hepatic homeostasis.

Viewed in this way, exercise initiates metabolic remodeling through the SIRT1–SIRT3–SIRT6 axis and completes that adaptation through the regulatory functions of SIRT2, SIRT4, SIRT5, and SIRT7. If past work has focused primarily on the “engine” that drives metabolic output, future exercise physiology must also consider the braking and cooling systems-the mechanisms that stabilize and regulate the response. Only then does the full molecular rationale for how exercise restores hepatic function become clear.

## Data Availability

The original contributions presented in the study are included in the article/supplementary material, further inquiries can be directed to the corresponding author.
