# Correction: Exercise and the hepatic sirtuin network: rethinking the research focus on SIRT1, SIRT3, and SIRT6

**DOI:** 10.3389/fphys.2026.1851497

**Published:** 2026-05-05

**Authors:** Jun Woo Kwon

**Affiliations:** Sports Technology Laboratory, Department of Physical Education, Seoul National University, Seoul, Republic of Korea

**Keywords:** exercise, hepatic metabolism, mitochondrial function, NAFLD, sirtuins

A correction has been made to the section **Generative AI statement:**

“The author acknowledges the use of generative artificial intelligence tools for limited language-related assistance, specifically for grammar checking and correction of typographical errors, and for the preparation of one figure. All scientific content, interpretations, and conclusions were conceived, written, and verified entirely by the author.”

The original version of this article has been updated.

